# Anti-Cancer Drug Solubility Development within a Green Solvent: Design of Novel and Robust Mathematical Models Based on Artificial Intelligence

**DOI:** 10.3390/molecules27165140

**Published:** 2022-08-12

**Authors:** Bader Huwaimel, Ahmed Alobaida

**Affiliations:** 1 Department of Pharmaceutical Chemistry, College of Pharmacy, University of Hail, Hail 81442, Saudi Arabia; 2 Depaertmen of Pharmaceutics, College of Pharmacy, University of Hail, Hail 81442, Saudi Arabia

**Keywords:** pharmaceutical industry, supercritical CO_2_, drug solubility, predictive models

## Abstract

Nowadays, supercritical CO_2_(SC-CO_2_) is known as a promising alternative for challengeable organic solvents in the pharmaceutical industry. The mathematical prediction and validation of drug solubility through SC-CO_2_ system using novel artificial intelligence (AI) approach has been considered as an interesting method. This work aims to evaluate the solubility of tamoxifen as a chemotherapeutic drug inside the SC-CO_2_ via the machine learning (ML) technique. This research employs and boosts three distinct models utilizing Adaboost methods. These models include K-nearest Neighbor (KNN), Theil-Sen Regression (TSR), and Gaussian Process (GPR). Two inputs, pressure and temperature, are considered to analyze the available data. Furthermore, the output is Y, which is solubility. As a result, ADA-KNN, ADA-GPR, and ADA-TSR show an R^2^ of 0.996, 0.967, 0.883, respectively, based on the analysis results. Additionally, with MAE metric, they had error rates of 1.98 × 10^−6^, 1.33 × 10^−6^, and 2.33 × 10^−6^, respectively. A model called ADA-KNN was selected as the best model and employed to obtain the optimum values, which can be represented as a vector: (X1 = 329, X2 = 318.0, Y = 6.004 × 10^−5^) according to the mentioned metrics and other visual analysis.

## 1. Introduction

The discovery of novel drug molecules followed by their introduction into clinical trials is considered as the main goal of the drug development industry for increasing the efficiency and reducing the side effects of drugs [1,2,3,4]. Solubility is one of the main parameters that influence drug efficiency [5,6]. Low solubility is considered as the most important challenge towards the formulation of novel chemical entities [7]. Various techniques can be used to improve drug solubility, such as physical modification (i.e., nanosuspension), chemical modification (i.e., complexation and salt formation), and miscellaneous procedures (i.e., supercritical fluids (SCFs) process and solubilizers) [8,9,10,11].

SCFs (especially supercritical CO_2_ (SC-CO_2_)) have been recently identified as a promising alternative for challengeable organic solvents. The emergence of remarkably positive points such as cost-effectiveness, inert nature, environmentally friendly, excellent chemical affinity in almost all organic solvents, safety of application and non-toxic characteristic has improved the tendency of researchers to apply them in pharmacology [12,13,14]. Additionally, the modulation of two momentous properties of CO_2_ including density and solvent power is feasible by the alteration of operational pressure/temperature and true control of the process kinetics [15,16,17,18].

The development of predictive models to estimate the solubility of various types of drugs in real conditions has been an interesting topic. Artificial intelligence (AI) approach is known as a robust and efficient approach to mathematically predict the results in various scientific scopes, such as nanotechnology, separation, extraction, chemical reactors, and transport phenomena [19,20,21,22,23].

Machine learning (ML) is a set of techniques and tools that uses data to create a mathematical model to make predictions or perform analysis, and it is critical in artificial intelligence [24,25]. ML approaches are progressively replacing computational methods in scientific domains. ML models may now investigate any problem with several input features and at least one target. These models extract inputs–outputs relationships using various strategies [26,27,28].

Boosting is a subtype of ensemble techniques that integrate the outcomes of several weak estimators to build a robust estimator. Boosting makes the usage of weak estimators applying a sequential logic, which implies the results of each weak estimate the influence of the following estimate. AdaBoost [29], in particular, is a representative boosting learning method that generates weak estimators gradually utilizing reweighted training data.

In recent years, GPR has gained popularity as a data-driven modeling tool. GPR’s popularity stems in part from its theoretical connection to Bayesian nonparametric statistics, infinite neural networks, kernel approaches in machine learning, and spatial statistics [30,31].

If the target data are numeric and continuous, neighbors-based regression such as KNN can be used. A query point’s label is determined by averaging the labels of its nearest neighbors [32].

Theil-Sen Regression is another weak estimator is used here. Compared to Ordinary Least Squares (OLS), Theil-Sen Regressor has a comparable asymptotic efficiency and is an unbiased estimate. Since it makes no assumptions about the underlying distribution of the data, Theil-Sen is non-parametric in comparison to OLS. Theil-Sen can withstand outliers more effectively [33,34].

The main novelty of this paper is to predict the optimized value of tamoxifen solubility in an SC-CO_2_ system via the ML approach. To achieve this, three ML-based predictive models including K-nearest Neighbor (KNN), Theil-Sen Regression (TSR), and Gaussian Process (GPR) were developed. The comparison of the models showed the fact that ADA-KNN is the most accurate and general model due to more proximity of points with actual test and train data lines and greater R^2^ value.

## 2. Dataset

In this research, a small dataset containing two inputs composed of X1 = P (bar) and X2 = T (K) and the only possible output is Y = solubility was applied. There are only 32 data points that were taken from the literature, and they performed the analysis for the pressure of 120–400 bar and temperature of 308–338 K [35]. The entire dataset is displayed in Table 1.

## 3. Methodology

### 3.1. Base Models

The first base model is a kernel-based and non-parametric method, Gaussian process regression (GPR). GPR focused on statistical learning theory and Bayesian models. When used in conjunction with the mean function, a kernel can be used to explain the covariance function of a Gaussian random variable. The GPR’s capacity to generalize well, particularly when working with minor data sets, is one of its most significant advantages [36,37,38]. When constructing a GPR model, the following equation is assumed to be true for an output *Y*:

(1) Y=f(X)+ξ

f(X) illustrates the underlying function, *X* as input of the training data, *X*_∗_ as test subset, and ξ~N(0, σ^2^) as the error. The error variance σ^2^ is calculated based on the input vector. The previous joint distribution of the actual target *Y* and the expected target *y* are [39,40]:

(2) $$Y\sim N\left(0,K\left(X,X\right)+\sigma^{2}I\right)$$

(3) $$\left[\begin{array}{c} Y\\ y \end{array}\right]\sim N\left(0,\left[\begin{array}{cc} K\left(X,X\right)+\sigma^{2}I & K\left(X,X_{*}\right)\\ K\left(X_{*},X\right) & K\left(X_{*},X_{*}\right) \end{array}\right]\right)=N\left(\left[\begin{array}{cc} K & K_{*}^{T}\\ K_{*} & K_{**} \end{array}\right]\right)$$

*K* = (*k_ij_*) as the covariance kernel matrix of the train subset in which the elements measure the relation between *X_i_* and *X_j_* through *k*. *K*_∗_ stands for the covariance matrix between the test and train subsets, and *K*_∗∗_ indicates the covariance matrix of the test subset [36,41]. The posterior distribution (in Bayesian analysis, reflects information about uncertain quantities) of *y* is shown in Equations (4)–(6):

(4) $$y|Y\sim N\left(\bar{y},\sigma_{y}^{2}\right)\;$$

(5) $$\bar{y}=K_{*}K^{-1}Y\;$$

(6) $$\sigma_{y}^{2}=K_{**}-K_{*}K^{-1}K_{*}^{T}\;$$

The other base models are K-nearest neighbor regression (KNN). The KNN regressor learns by comparison of the identified test examples to the training set [42]. $T=\left\{\left(x_{1},y_{1}\right),\ldots ,\;\left(x_{N},y_{N}\right)\right\}$ represent the training data with a parameter of distance *d*. $x_{i}=\left(x_{i1},\;x_{i2},\;x_{i3},\ldots ,x_{im}\right)$ represent the *i*-th sample indicates with *m* input features and its target output *y_i_*. Additionally, *N* represent the count of examples. It must calculate the *d_i_* between a test instance *x* and any sample *x_i_* *∈ T* and sort the *d_i_* distance by its value for a test sample *x*. If *d_i_* is in the *i*-th place, the instance of x matches di, which is called the *i*-th nearest neighbor, or *NN_i_(x)*, and its target is called *y_i_(x)*. Lastly, the estimation $\hat{y}$ of input instance *x* denotes the average of the prediction of *k*-nearest neighbors to *x* ($\hat{y}=\frac{1}{k}\overset{k}{\underset{i=1}{\sum }}y_{i}\left(x\right))$. KNN regression algorithm can be summarized in the following steps [43]:

- Inputs: training samples $\left\{x_{i},y_{i}\right\},\;x_{i}$: input features, $y_{i}$: real-valued output, testing point *x* to predict
- Algorithm:
- Calculate distance $D\left(x,x_{i}\right)$ to every training example $x_{i}$
- Select k closet examples $x_{i1}\ldots x_{ik}$ and their outputs $y_{i1}\ldots y_{ik}$
- Output:

(7) $$\hat{y}=f\left(x\right)=\frac{1}{K}\overset{k}{\underset{j=1}{\sum }}y_{i_{j}}$$

The third base model is Theil-Sen Regression. The model is estimated in Theil-Sen regression by computing the slopes and intercepts of a subset of all feasible solutions of *p* subsample points. When an intercept is fitting, *p* must be bigger than or equal to number of features + 1. The spatial median of these slopes and intercepts is then used to define the final slope and intercept.

The trend slopes were estimated using the Theil-Sen (TSR) estimator [44], which was chosen since it is better than raw linear regression approaches in evaluating trend slopes in the existence of outliers in data [45].

The initial phase in calculating the TSR predictor is to determine the *Q_i_* value given *N* pairs of data [44]:

(8) $$Q_{i}=\frac{x_{j}-x_{k}}{j-k}\;i\in \left\{1,2,\ldots ,N\right\}\;$$

*x_j_*, *x_k_* are the data point vectors.

If only one datum is existed, then $N=\frac{n\left(n–1\right)}{2}$. Additionally, n is the count of vectors. If there are many observed data in several vectors, then $N<\frac{n\left(n–1\right)}{2}$, *n* is the count of observed vectors.

Then, the TSR predictor is calculated as the median *Q_med_* of the *N* values of *Q_i_*, sorted in (minimum, maximum) interval [44]:

(9) $$Q_{med}=\{\begin{array}{c} Q_{\frac{\left(N+1\right)}{2}}\;when\;N\;is\;odd\\ \begin{array}{c} \frac{Q_{\frac{N}{2}+}Q_{\frac{\left(N+1\right)}{2}}}{2}\;when\;N\;is\;even \end{array} \end{array}\;\;$$

The sign of *Q_med_* shows the trend behavior, and its value shows the magnitude of the trend.

### 3.2. AdaBoost

Adaboost [46] is the most well-known boosting model, and it was initially employed to address the classification issue. Freund [29] then presented the Adaboost.R to handle real-valued regression problems. Additionally, drucker [47] solved the regression problem using the updated Adaboost.R2 model, with amazing results.

The data sample weights are set to zero. The initial iteration trains a weak learner, and the instance weights are adjusted based on the training outcomes. The adjusted weights are used to train the next weak learner. Each iteration, the weights of the instances estimated incorrectly (with a high error) in the previous iteration are increased, while the weights of the instances estimated correctly (near expected value) in the previous iteration are decreased. The influence of hard-to-predict instances becomes increasingly substantial as the number of iterations grows; after each iteration, the weak learner concentrates more on samples that were previously estimated poorly. The final prediction outcome is established by a weak learner’s weighted vote. Any machine learning regression technique may be used to choose the weak learner in AdaBoost regression [48,49,50]. In this study, we used three models of previous section as weak learners distinctly.

## 4. Results

We employed grid search to find the optimal hyper-parameters of these models and obtained the final configuration of each model. MAE and R^2^ are two metrics that were used to evaluate the performance of the model that were calculated using Equations (10) and (11) [51,52].

(10) $$\mathrm{MAE}=\frac{1}{n}\sum^{\;}\left|{\overset{{}^{}}{x}}_{i}^{t+1}-x_{i}^{t+1}\right|$$

(11) R2=1−∑ (x^it+1−xit+1)2∑ (x¯it+1−xit+1)2 

In these equations, $x_{i}^{t+1}$ is the estimated value, $x_{i}^{t+1}$ is the observed value, and *n* is the quantity of examples.

**Figure 1:**
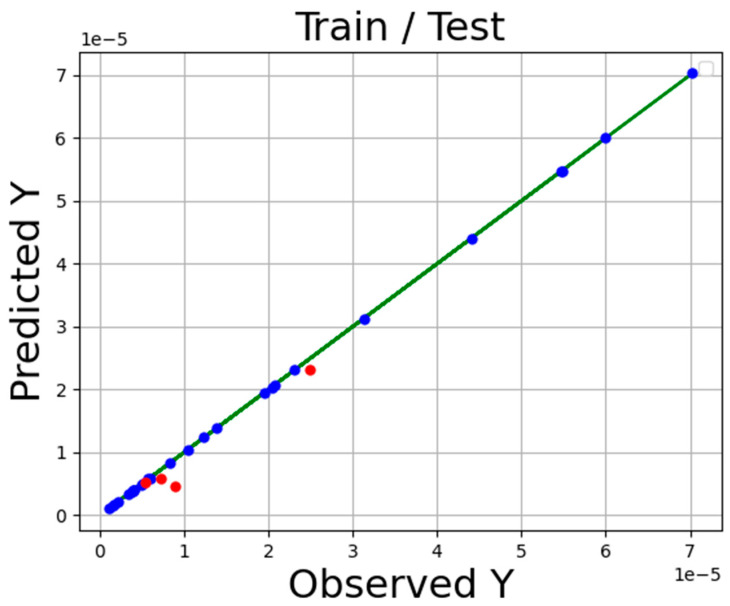
Fitting chart for ADA-KNN.

**Figure 2:**
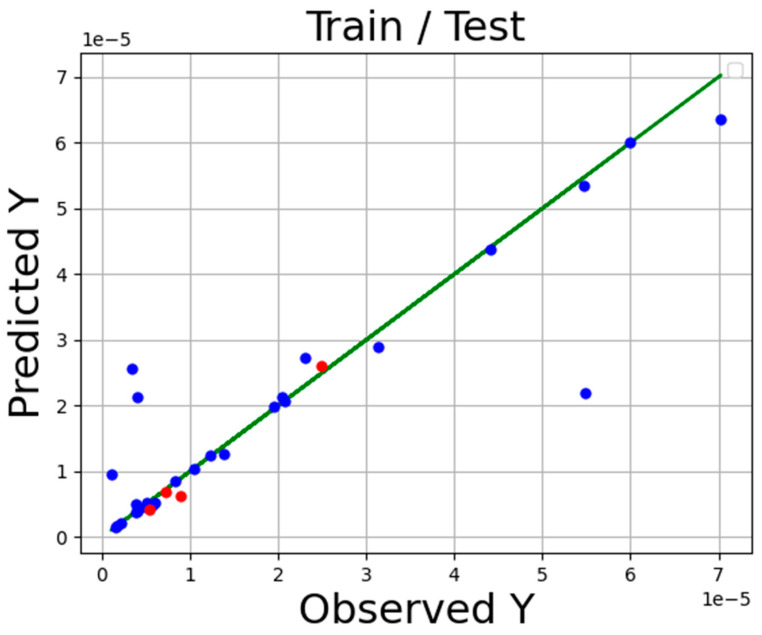
Fitting chart for ADA-GPR.

**Figure 3:**
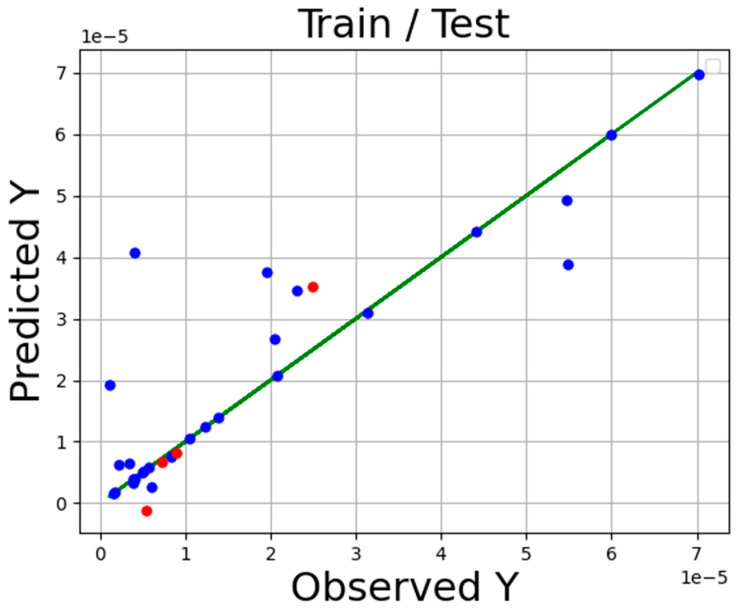
Fitting chart for ADA-TSR.

The accuracy of the final models is presented in Table 2 Additionally, the comparison of expected and estimated values of tamoxifen solubility in SC-CO_2_ system via ADA-KNN, ADA-GPR, and ADA-TSR models is shown in Figure 1, Figure 2 and Figure 3. In these diagrams, the green line is the actual data line, and the point is predicted values blue for train sunset and red for test subset. Comparing these three charts proves that the ADA-KNN is the most general and appropriate model since the points are near actual test and train data lines.

**Figure 4:**
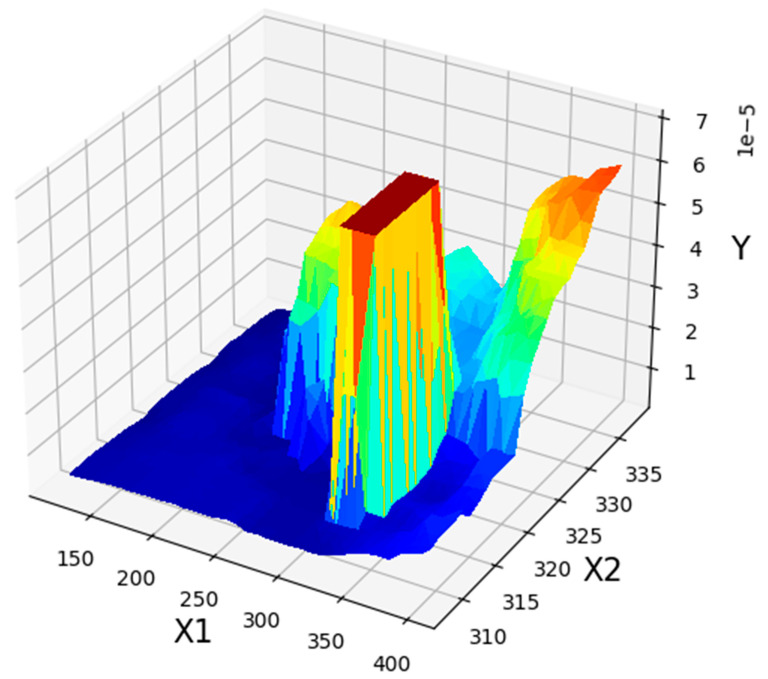
Three-dimensional illustration of pressure (X1), temperature (X2), and solubility (Y).

**Figure 5:**
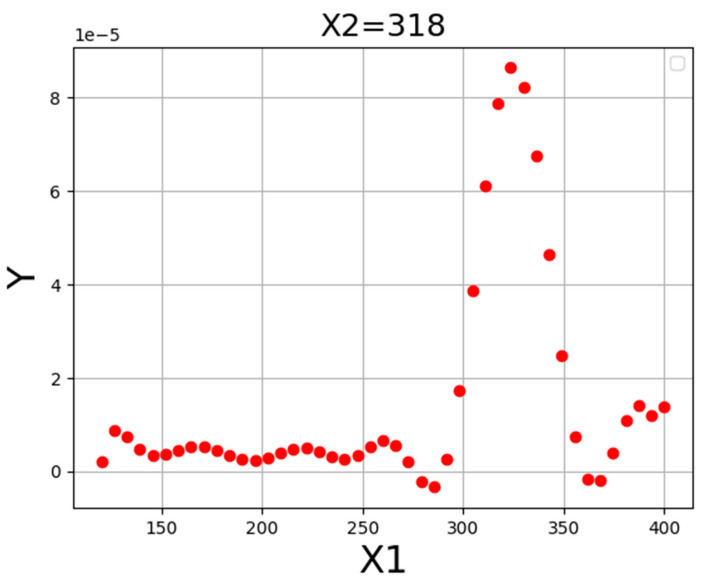
Tendency of X1.

**Figure 6:**
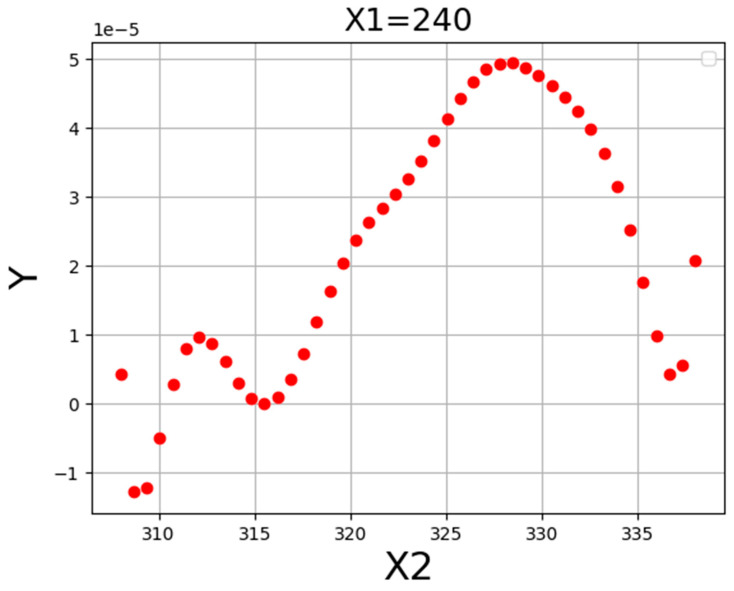
Tendency of X2.

Figure 4 illustrates the three-dimensional projection to demonstrate the final results of the ADA-KNN mathematical model to measure the impacts of input parameters (pressure and temperature) on drug solubility at the same time. Furthermore, two-dimensional depictions to individually evaluate the effects of pressure and temperature on the values of tamoxifen solubility in SC-CO_2_ system are shown in Figure 5 and Figure 6. It can be seen from the figures that pressure has positive effect on the solubility value of drugs in the SC-CO_2_ fluid due to increasing the density of SCFs owing to modify the molecular compaction. If the value of density increases, the solvating capability of solvent increases significantly and, the solubility of drug in SC-CO_2_ increases. The effect of temperature on drug solubility is paradoxical. In one side, increment of temperature improves the pressure sublimation of solvent, which is a positive phenomenon in increasing the solubility of the drug inside SCFs. On the other side, the increase in temperature reduces the density of solvent, which considerably deteriorates the solvating power and consequently solubility amount of drug. Considering the abovementioned explanations, the net impacts of the sublimation pressure and density can determine the favorable/unfavorable role of temperature on the solubility. The evaluation of figures illustrates the emergence of a cross-over pressure in the isotherms. At the pressures over than cross-over pressure, an increase in temperature improves the drug solubility because of the greater effect of sublimation pressure compared to density. For the pressures lower than the cross-over pressure, increasing the temperature, decrement in the solvent density overcomes the effect of pressure sublimation and as a result, and decreases the tamoxifen solubility in SC-CO_2_ fluid [35]. Based on the presented results of Table 3, the pressure and temperature at 329 bar and 318 K, respectively, were considered as the optimum pressure and temperature for reaching the maximum amount of tamoxifen solubility.

## 5. Conclusions

In this research work, three new models were compared through machine learning to estimate and validate the solubility of tamoxifen in supercritical CO_2_. The Adaboost method was applied to improve these three different models, including KNN, GPR and TSR, and the results are promising. According to the analysis, the R^2^ of the ADA-KNN, ADA-GPR, and ADA-TSR models were 0.996, 0.967, and 0.883, respectively. The MAE error rates for these three models were 1.98 × 10^−6^, 1.33 × 10^−6^, and 2.33 × 10^−6^, respectively. An ADA-KNN model was selected as the best model, and it was applied to optimize the values using these metrics (X1 = 329, X2 = 318.0, Y = 6.004 × 10^−5^) and some visual analysis.

## Figures and Tables

**Table 1:** Dataset.

No.	X1 = P (bar)	X2 = T (K)	Y (Solubility/Mole Fraction)
1	120	308	4 × 10^−6^
2	160	308	4.94 × 10^−6^
3	200	308	5.49 × 10^−6^
4	240	308	5.96 × 10^−6^
5	280	308	3.99 × 10^−6^
6	320	308	3.88 × 10^−6^
7	360	308	8.38 × 10^−6^
8	400	308	1.24 × 10^−5^
9	120	318	2.15 × 10^−6^
10	160	318	5.79 × 10^−6^
11	200	318	8.95 × 10^−6^
12	240	318	7.27 × 10^−6^
13	280	318	3.40 × 10^−6^
14	320	318	7.03 × 10^−5^
15	360	318	4.01 × 10^−6^
16	400	318	1.39 × 10^−5^
17	120	328	1.79 × 10^−6^
18	160	328	5.13 × 10^−6^
19	200	328	1.05 × 10^−6^
20	240	328	5.48 × 10^−5^
21	280	328	2.31 × 10^−5^
22	320	328	2.04 × 10^−5^
23	360	328	2.50 × 10^−5^
24	400	328	4.41 × 10^−5^
25	120	338	1.52 × 10^−5^
26	160	338	3.84 × 10^−6^
27	200	338	1.05 × 10^−5^
28	240	338	2.08 × 10^−5^
29	280	338	3.13 × 10^−5^
30	320	338	1.95 × 10^−5^
31	360	338	5.47 × 10^−5^
32	400	338	6.0 × 10^−5^

**Table 2:** Output.

Models	MAE	R^2^
ADA-KNN	1.98 × 10^−6^	0.996
ADA-GPR	1.33 × 10^−6^	0.967
ADA-TSR	2.33 × 10^−6^	0.883

**Table 3:** Modified parameters applying maximum response.

X1 = P (bar)	X2 = T (K)	Y (Solubility)
329	318.0	7.03 × 10^−5^

## Data Availability

All data are available within the published paper.

## References

[1] Faqi A.S. (2016). A Comprehensive Guide to Toxicology in Nonclinical Drug Development.

[2] Brun R., Don R., Jacobs R.T., Wang M.Z., Barrett M.P. (2011). Development of novel drugs for human African trypanosomiasis. Future Microbiol..

[3] Martell R.E., Brooks D.G., Wang Y., Wilcoxen K. (2013). Discovery of novel drugs for promising targets. Clin. Ther..

[4] Mirhaji E., Afshar M., Rezvani S., Yoosefian M. (2018). Boron nitride nanotubes as a nanotransporter for anti-cancer docetaxel drug in water/ethanol solution. J. Mol. Liq..

[5] Savjani K.T., Gajjar A.K., Savjani J.K. (2012). Drug solubility: Importance and enhancement techniques. Int. Sch. Res. Not..

[6] Gorain B., Pandey M., Choudhury H., Jain G.K., Kesharwani P. (2021). Dendrimer for solubility enhancement. Dendrimer-Based Nanotherapeutics.

[7] Williams H.D., Trevaskis N.L., Charman S.A., Shanker R.M., Charman W.N., Pouton C.W., Porter C.J.H. (2013). Strategies to address low drug solubility in discovery and development. Pharmacol. Rev..

[8] Vimalson D.C. (2016). Techniques to enhance solubility of hydrophobic drugs: An overview. Asian J. Pharm..

[9] Das B., Baidya A.T., Mathew A.T., Yadav A.K., Kumar R. (2022). Structural modification aimed for improving solubility of lead compounds in early phase drug discovery. Bioorganic Med. Chem..

[10] Bagade O., Kad D.R., Bhargude D.N., Bhosale D.R., Kahane S.K. (2014). Consequences and impose of solubility enhancement of poorly water soluble drugs. Res. J. Pharm. Technol..

[11] Cao M., Yoosefian D.W.M., Sabaei S., Jahani M. (2020). Comprehensive study of the encapsulation of Lomustine anticancer drug into single walled carbon nanotubes (SWCNTs): Solvent effects, molecular conformations, electronic properties and intramolecular hydrogen bond strength. J. Mol. Liq..

[12] Girotra P., Singh S.K., Nagpal K. (2013). Supercritical fluid technology: A promising approach in pharmaceutical research. Pharm. Dev. Technol..

[13] Macnaughton S.J., Kikic I., Foster N.R., Alessi P., Cortesi A., Colombo I. (1996). Solubility of anti-inflammatory drugs in supercritical carbon dioxide. J. Chem. Eng. Data.

[14] Zhou M., Ni R., Zhao Y., Huang J., Deng X. (2021). Research progress on supercritical CO_2_ thickeners. Soft Matter.

[15] Baldino L., Cardea S., Reverchon E. (2017). Biodegradable membranes loaded with curcumin to be used as engineered independent devices in active packaging. J. Taiwan Inst. Chem. Eng..

[16] Su W., Zhang H., Xing Y., Li X., Wang J., Cai C. (2021). A bibliometric analysis and review of supercritical fluids for the synthesis of nanomaterials. Nanomaterials.

[17] Baldino L., della Porta G., Reverchon E. (2017). Supercritical CO_2_ processing strategies for pyrethrins selective extraction. J. CO2 Util..

[18] Yoosefian M., Sabaei S., Etminan N. (2019). Encapsulation efficiency of single-walled carbon nanotube for Ifosfamide anti-cancer drug. Comput. Biol. Med..

[19] Zhu H., Zhu L., Sun Z., Khan A. (2021). Machine learning based simulation of an anti-cancer drug (busulfan) solubility in supercritical carbon dioxide: ANFIS model and experimental validation. J. Mol. Liq..

[20] Öztürk A.A., Gündüz A.B., Ozisik O. (2018). Supervised machine learning algorithms for evaluation of solid lipid nanoparticles and particle size. Comb. Chem. High Throughput Screen..

[21] Staszak M. (2020). Artificial intelligence in the modeling of chemical reactions kinetics. Phys. Sci. Rev..

[22] Wang X., Luo L., Xiang J., Zheng S., Shittu S., Wang Z., Zhao X. (2021). A comprehensive review on the application of nanofluid in heat pipe based on the machine learning: Theory, application and prediction. Renew. Sustain. Energy Rev..

[23] Lazzús J.A., Cuturrufo F., Pulgar-Villarroel G., Salfate I., Vega P. (2017). Estimating the temperature-dependent surface tension of ionic liquids using a neural network-based group contribution method. Ind. Eng. Chem. Res..

[24] Murphy K.P. (2012). Machine Learning: A Probabilistic Perspective.

[25] Mitchell T.M. (2006). The Discipline of Machine Learning.

[26] El Naqa I., Murphy M.J. (2015). What is machine learning?. Machine Learning in Radiation Oncology.

[27] Goodfellow I., Bengio Y., Courville A. (2016). Machine learning basics. Deep. Learn..

[28] Shehadeh A., Alshboul O., Al Mamlook R.E., Hamedat O. (2021). Machine learning models for predicting the residual value of heavy construction equipment: An evaluation of modified decision tree, LightGBM, and XGBoost regression. Autom. Constr..

[29] Freund Y., Schapire R.E. (1997). A decision-theoretic generalization of on-line learning and an application to boosting. J. Comput. Syst. Sci..

[30] Rasmussen C.E. (2003). Gaussian processes in machine learning. Summer School on Machine Learning.

[31] Shi J.Q., Choi T. (2011). Gaussian Process Regression Analysis for Functional Data.

[32] Masegosa R.A., Armañanzas R., Abad-Grau M.M., Potenciano V., Moral S., Larrañaga P., Bielza C., Matesanz F. (2015). Discretization of Expression Quantitative Trait Loci in Association Analysis Between Genotypes and Expression Data. Curr. Bioinform..

[33] Wilcox R. (1998). A note on the Theil-Sen regression estimator when the regressor is random and the error term is heteroscedastic. Biom. J..

[34] Ohlson J.A., Kim S. (2015). Linear valuation without OLS: The Theil-Sen estimation approach. Rev. Account. Stud..

[35] Pishnamazi M., Zabihi S., Jamshidian S., Borousan F., Hezave A.Z., Shirazian S. (2020). Thermodynamic modelling and experimental validation of pharmaceutical solubility in supercritical solvent. J. Mol. Liq..

[36] Williams C.K., Rasmussen C.E. (1996). Gaussian Processes for Regression.

[37] Rasmussen C.E. (1997). Evaluation of Gaussian Processes and Other Methods for Non-Linear Regression.

[38] Taherdangkoo R., Yang H., Akbariforouz M., Sun Y., Liu Q., Butscher C. (2021). Gaussian process regression to determine water content of methane: Application to methane transport modeling. J. Contam. Hydrol..

[39] Alghamdi A.S., Polat K., Alghoson A., Alshdadi A.A., Abd El-Latif A.A. (2020). Gaussian process regression (GPR) based non-invasive continuous blood pressure prediction method from cuff oscillometric signals. Appl. Acoust..

[40] Cheng M., Prayogo D. (2016). Optimizing Biodiesel Production from Rice Bran Using Artificial Intelligence Approaches.

[41] Williams C.K., Barber D. (1998). Bayesian classification with Gaussian processes. IEEE Trans. Pattern Anal. Mach. Intell..

[42] Cover T. (1968). Estimation by the nearest neighbor rule. IEEE Trans. Inf. Theory.

[43] Song Y., Liang J., Lu J., Zhao X. (2017). An efficient instance selection algorithm for k nearest neighbor regression. Neurocomputing.

[44] Sen P.K. (1968). Estimates of the regression coefficient based on Kendall’s tau. J. Am. Stat. Assoc..

[45] Caloiero T., Aristodemo F., Ferraro D.A. (2022). Annual and seasonal trend detection of significant wave height, energy period and wave power in the Mediterranean Sea. Ocean. Eng..

[46] Freund Y., Schapire R.E. (1996). Experiments with a new boosting algorithm. Proceedings of the Thirteenth International Conference on International Conference on Machine Learning.

[47] Drucker H. (1997). Improving regressors using boosting techniques. Proceedings of the Fourteenth International Conference on Machine Learning.

[48] Dargahi-Zarandi A., Hemmati-Sarapardeh A., Shateri M., Menad N.A., Ahmadi M. (2020). Modeling minimum miscibility pressure of pure/impure CO2-crude oil systems using adaptive boosting support vector regression: Application to gas injection processes. J. Pet. Sci. Eng..

[49] Wu Q., Burges C.J.C., Svore K.M., Gao J. (2010). Adapting boosting for information retrieval measures. Inf. Retr..

[50] Ying C., Miao Q., Liu J., Gao L. (2013). Advance and prospects of AdaBoost algorithm. Acta Autom. Sin..

[51] Botchkarev A. (2018). Evaluating Performance of Regression Machine Learning Models Using Multiple Error Metrics in Azure Machine Learning Studio. https://papers.ssrn.com/sol3/papers.cfm?abstract_id=3177507.

[52] Kumar S., Mishra S., Singh S.K. (2020). A machine learning-based model to estimate PM2. 5 concentration levels in Delhi’s atmosphere. Heliyon.

